# A 10-Week Multimodal Nutrition Education Intervention Improves Dietary Intake among University Students: Cluster Randomised Controlled Trial

**DOI:** 10.1155/2013/658642

**Published:** 2013-08-28

**Authors:** Mohd Razif Shahril, Wan Putri Elena Wan Dali, Pei Lin Lua

**Affiliations:** ^1^School of Nutrition and Dietetics, Faculty of Medicine and Health Sciences, Universiti Sultan Zainal Abidin, Jalan Sultan Mahmud, 20400 Kuala Terengganu, Terengganu, Malaysia; ^2^Centre for Community Development and Quality of Life, Universiti Sultan Zainal Abidin, Jalan Sultan Mahmud, 20400 Kuala Terengganu, Terengganu, Malaysia

## Abstract

The aim of the study was to evaluate the effectiveness of implementing multimodal nutrition education intervention (NEI) to improve dietary intake among university students. The design of study used was cluster randomised controlled design at four public universities in East Coast of Malaysia. A total of 417 university students participated in the study. They were randomly selected and assigned into two arms, that is, intervention group (IG) or control group (CG) according to their cluster. The IG received 10-week multimodal intervention using three modes (conventional lecture, brochures, and text messages) while CG did not receive any intervention. Dietary intake was assessed before and after intervention and outcomes reported as nutrient intakes as well as average daily servings of food intake. Analysis of covariance (ANCOVA) and adjusted effect size were used to determine difference in dietary changes between groups and time. Results showed that, compared to CG, participants in IG significantly improved their dietary intake by increasing their energy intake, carbohydrate, calcium, vitamin C and thiamine, fruits and 100% fruit juice, fish, egg, milk, and dairy products while at the same time significantly decreased their processed food intake. In conclusion, multimodal NEI focusing on healthy eating promotion is an effective approach to improve dietary intakes among university students.

## 1. Introduction

The transition from adolescence to young adulthood which is mostly spent at colleges or universities is gaining recognition as an important time for health promotion and disease prevention [1]. This is the critical time during which young people establish independence and adopt lasting health behaviour patterns. Although once considered to be an age of optimal health and well-being, it is well documented that university students nowadays have poor dietary habits [2, 3]. University students often fail to meet recommended target for fruits and vegetables (FV) [4, 5], whole grain, milk, and dairy products [6, 7] compared to their adolescence years. They also tend to skip meals especially breakfast [8, 9] and have higher consumption of fast food, snacks, and soft drinks [10–12]. 

Poor eating habits in earlier stage of life have been directly linked to serious health consequences later in life such as osteoporosis, obesity, hyperlipidemia, and diabetes [13, 14]. Therefore early interventions are needed to improve health behaviours in this age group [15]. Currently only a few nutrition education interventions (NEI) have targeted college or university students relative to interventions designed for children and elderly [16]. Designing NEI for university students is challenging as conventional methods alone in delivering NEI might not attract this age group. A multimodal NEI by combining both latest technology and conventional method might become a viable means in disseminating NEI. Previous researches have shown that class-based NEI [17] and brochures [18] improved dietary habits of university students. When these conventional methods were used alone, they appeared to be successful only in increasing nutrition knowledge and not changing dietary habits remarkably. 

In this fast-moving world, intervention delivery through text messaging may be more cost effective than other telephone or print-based tools. Text messaging, otherwise known as short message service (SMS), has become an important modality for mobile communication and numerous studies proved that text messaging has positive short-term behavioral outcomes [19, 20]. Improving nutrition knowledge, eating behaviours, and dietary practices through multimodal NEI may help to prevent or mitigate noncommunicable diseases. However to our knowledge, a multimodal NEI using text messaging, brochures, and conventional lecture for university students has not yet been empirically tested. The purpose of this study was to evaluate the effectiveness of implementing multimodal NEI to improve dietary intake among university students. The present intervention is unique in combining the use of conventional NEI tools, that is, lecture and brochures with text messaging as reminder for intervention reinforcement.

## 2. Methodology

### 2.1. Participants

**Figure 1:**
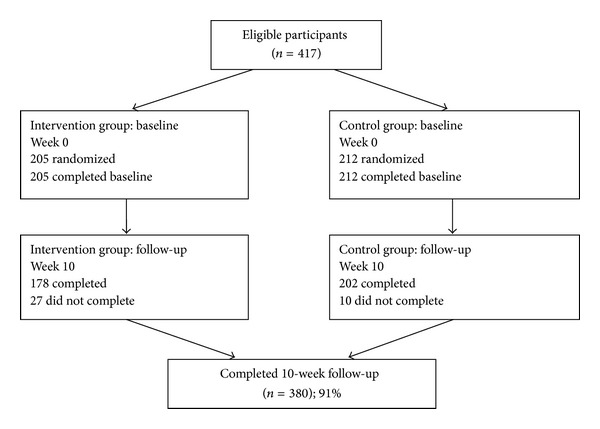
Consort diagram.

This longitudinal study was carried out in four public universities in East Coast of Malaysia starting September 2011 until February 2012. The lists of all available classes (also called as clusters) were gathered from heads of department from each university. From these lists, a total of 16 classes were selected randomly using simple random sampling to represent the target population of this study. Included participants were Malaysian university students aged between 18 and 24 years; actively using a mobile telephone; first or second year diploma or degree from management studies; generally healthy and able to read, write, speak, and understand Malay or English language. Respondents were excluded if their age was below or above the stated age (<18 years or >24 years); did not have mobile phones; were in the final year and in other studies; have been diagnosed with any diseases and were unable to read, write, speak, or understand Malay or English. All randomly selected clusters were then randomised into intervention group (IG) and control group (CG) by drawing sealed envelopes containing group assignment. At the end of this study, 417 university students agreed to participate (IG = 205, CG = 212). However, only 380 students completed the entire study (IG = 178, CG = 202) (Figure 1).

### 2.2. Measure

Measures were taken at baseline and after 10 weeks of NEI. At baseline, all students initially completed a personal information form, which comprised demographic questions recording their gender, living arrangement, academic year, funding status, and body mass index (BMI). The BMI (kg/m^2^) was calculated using the individual's height and weight and classified according to the Asian population categorisation [22]. The measurement of dietary intake was conducted using diet history. A diet history is a structured interview method consisting of questions about habitual intake of foods from the core (e.g., cereals, meat and alternatives, fruit and vegetables, dairy, and other foods) food groups in the last seven days [23]. The design used in this study was based on the local dietary habits. Participants were interviewed about all details including type of food, cooking methods, and estimated portion size with local household measurement of food and beverages consumed for the past week to estimate their dietary intakes. Frequency of intake was also recorded with response options range from once a week to several times a day for each food. The estimated amount of food consumed was then converted into grams and was analysed by using Nutritionist Pro (Axxya Systems, USA). To ensure the validity of data used in this study, only those with ratio of 1.2 to 1.8 for Energy Intake per Estimated Basal Metabolism Rate which indicates normal reporting were included. This eliminates misreporting issue among study participants. 

### 2.3. Procedure

Four hundred and seventeen students were randomized according to their cluster to one of these groups: (1) IG (*n* = 205); and (2) CG (*n* = 212). Once randomisation was complete, research assistants (RAs) arranged a meeting with all participants according to their respective clusters to gather baseline data prior to the start of intervention. During the first session, all participants, both from IG and CG, signed the IRB approved consent form and then completed the baseline dietary intake assessment. Participants in IG scheduled a second 1.5 hours meeting in a week time from baseline, during which they received a conventional nutrition lecture by a nutrition expert and three sets of brochures as take home messages. They also received one text message every five days starting from baseline until end of week 10. A total of 13 text messages were delivered for each participant in IG during intervention. CG participants received no intervention and were instructed to maintain their normal dietary habits and daily activity. Ten weeks after completing baseline session, all participants were called back according to their clusters to complete the follow-up assessment. Participants were not given any incentives in return for their involvement in this study.

### 2.4. Intervention

The NEI employed was based on the latest Malaysian Dietary Guidelines (MDG) [24] which comprised 13 out of 14 nutrition key messages (Table 1). Messages which deliberated on *Practise Exclusive Breastfeeding from Birth until Six Months and Continue to Breastfeed until Two Years of Age *were excluded due to their irrelevance to the current participants who were young undergraduates who were mostly unmarried. All included messages were delivered through three modes: (1) conventional lecture, (2) brochures, and (3) text messaging. Malay language as the national language was used in delivering this multimodal NEI. Content validity and face validity of these multimodal NEI were initially evaluated by two qualified researchers experienced in nutrition and dietetics and were then pretested among 116 university students for clarity and readability as well as the overall content [25]. Subsequently, the contents in this multimodal NEI were modified based on the feedbacks obtained from respondents recruited during the pretest.

#### 2.4.1. Conventional Lecture

Conventional lectures were carried out in which all selected key messages in the guidelines were compiled into a 64-slide multimedia Microsoft PowerPoint presentation. The slides used were clearly visible for approximately 100 students with appropriate font sizes. Attractive graphics and suitable combination colours were additionally used to stimulate their interest on the topics delivered in a 1-hour session by the RA who has basic knowledge in food and nutrition. Question and answer session were conducted along the lecture for active participations of the audience. 

#### 2.4.2. Brochure

Brochures were designed as take home messages to enhance their understanding and memory after the lecture. These brochures contained key recommendations and how to achieve the recommendations for each key message through three different theme, namely, *Always Be Healthy!, Eat Moderately!, *and *Live the Future!*. The information was displayed on the coloured art papers in 35.8 cm × 25 cm-sized with four folded and printed double-sided. Pictorial graphics which include food pictures, cartoon pictures, and symbols were used to attract the readers. The text language was kept simple with black 12-font sized.

#### 2.4.3. Text Messaging

Text messaging developed in this study can be delivered to all forms of cellular telephone with a limitation of 152 characters for each text message. A total of 13 text messages based on the MDG [24] were delivered and designed to be sent once in every five days at 10 a.m. throughout the intervention period. Text messages were sent manually through the Mobile Nutritional Education System (MNES) which was developed by a mobile content and services provider based in Kuala Lumpur, Malaysia. Abbreviations were avoided to prevent misunderstanding of the information received. 

### 2.5. Ethical Approval

Ethical approval was granted by the Institute of Health Behavioral Research (IHBR), Clinical Research Centre (CRC), and Ministry of Health Research and Ethics Committee (MREC), Malaysia. Apart from that, permission to conduct the study in each participating university was also obtained from the Vice Chancellors and heads of department prior to data collection process. Permission to use the latest MDG was also approved by the Nutrition Division, Ministry of Health, Malaysia.

### 2.6. Statistical Analysis

Initial normality test was carried out utilizing the age and dietary intake as dependent variables. The overall outcomes complied with normality requirements in which the Kolmogorov-Smirnov statistics emerged as *P* > 0.05. The IG and CG were compared descriptively with respect to sociodemographic characteristics. All data analyses were performed using SPSS for Windows version 16.0. Analysis of covariance (ANCOVA) was utilised to examine the changes in dietary intakes from baseline to 10 weeks after intervention between IG and CG with potentially confounding factors (weight, waist, hip, and baseline readings) included as covariates. Adjusted effect sizes using Cohen's formula were also added (adjusted mean difference/*√*mean square error). The values of adjusted effect sizes were interpreted according to these scales: 0.20–0.49 = small; 0.50–0.79 = medium; ≥0.80 = large [26]. Significance was set a priori at *P* < 0.05.

## 3. Results

### 3.1. Participants' Characteristics

Average age of the respondents is 19.1 years (range = 18–24). For both groups, most of them were females; lived with friends, studied in the first year and their studies were funded by National Higher Education Fund (PTPTN) or Council of Trust for Indigenous People (MARA).  Table 2 summarises selected characteristics of study participants. Majority of the participants also have normal BMI range (CG = 50.5%; IG = 46.1%).

### 3.2. Changes on Dietary Intake

Unadjusted mean energy and nutrient intakes at baseline and after 10 weeks of intervention among IG and CG were presented in Table 3. Increased mean energy and carbohydrate intake were observed in both IG and CG participants while fat intake was decreased. Both IG and CG participants' intake of protein remained unchanged after a 10 week of intervention. Further analysis controlling for potential confounders such as weight, waist circumference, hip circumference, and baseline measures showed that after 10 weeks of intervention, energy intake among IG participants increased significantly compared to CG (*P* = 0.006) with an adjusted effect size of 0.28 (small effect). The percentage of carbohydrate, protein, and fat contribution to energy was unaffected after 10 weeks of multimodal NEI in this study as the predicted interaction between time and group was not significant. Other than that, IG possessed relatively better intakes in calcium, vitamin C, and thiamine compared to CG with the largest adjusted effect size in vitamin C (0.93). 

Table 4 presents dietary intake according to specific food groups at baseline and after 10 weeks of intervention among IG and CG. IG participants improved their fruit and 100% fruit juice, fish, egg, milk, dairy products and processed food intake pattern while their counterparts in CG remained with their baseline diet. After controlling for potential confounders, the results showed that intakes of fruits and 100% fruit juice, fish, egg, milk and dairy products were significantly increased in IG compared to CG after 10 weeks of multimodal NEI. Intake of processed foods decreased significantly over 10 week time in IG compared to CG. These changes were nonetheless still considered as a small change based on Cohen's interpretation except for changes on fruits and 100% fruit juices intake which showed a tremendous large adjusted effect size of 1.03.

## 4. Discussion

The present investigation examined the effectiveness of implementing multimodal NEI to promote healthy eating pattern among university students. A considerable amount of literature has reported that university students displayed unhealthy eating patterns during their varsity years [2–4, 6–12]. This study confirmed that these students, based on the baselines data, were generally not adhering to the local dietary guidelines especially for FV, fish, egg, nuts and legumes, milk, and dairy products. They were also practicing unhealthy eating habits with frequent consumption of processed foods and beverages with sweetened condensed milk. 

Exposure to a multimodal NEI using conventional lecture, three brochures as take-home messages, and periodical reminder through text messaging for 10 weeks among university students was found through this study to be beneficial in improving their food choices. No significant changes on macronutrient composition were observed in this study, although there was a potential for a decreasing trend of fat intake throughout the intervention. This was due to mean baseline macronutrient composition which was at near healthy range, except for higher fat intake (more than 30% of energy). However, energy intake was increased significantly after 10 weeks of multimodal NEI to meet the general requirement of 2000 kcal per day among IG participants who were majority female. It was also quite interesting for this study to have data on specific dietary intake pattern according to major food groups which was not presented by most previous studies [16]. 

Additionally, changes on cereal and cereal products were not observed in this study although emphasis has been given to increase whole grain intake through intervention using various modalities in the current study. It was noteworthy that whole grain products in this region were limited and expensive and this might be the reason for low adherence to the invention messages delivered on increasing whole grain intake among students who were known to have restricted budget. In a study conducted by researchers from Kent State University, an interactive introductory nutrition class (50-minute sessions 3 times a week) for a semester among university students helped to increase whole grain intake by almost three times higher than baseline intake [27]. This shows that lectures as a mean of NEI delivery might have potential to be an effective tool in increasing whole grain intake among college students provided the whole grain products were easily accessible to the target population. 

Fruits and 100% fruit juices consumption on the other hand had increased almost three times higher than baseline after 10-week intervention among IG participants in the current study compared to controls. Even though the recommended intake for fruits of two servings per day is not achieved, this finding demonstrated a significant improvement over baseline intake. Previously, a social-marketing campaign pilot study by distributing fresh fruit, 100% fruit juice, and fruit smoothie samples and information about fruit during a two months fruit fair to improve knowledge, attitudes, and fruit intake among community college students found a significant increase in fruit intake between pre- and posttest at the intervention campus [18]. Although the researchers from California State University addressed policy change to increase the accessibility of fruit on campus, most students did not achieve the minimum recommended daily two servings of fruit which might due to insufficient fund. On the other hand, a systemic review concluded that FV intake was increased by 0.1 to 1.4 serving per day in primary prevention interventions among healthy adults [28]. The same report found consistent positive effects in studies involving face-to-face education or counselling, but interventions using telephone contacts or computer-tailored information appeared to be a comparable alternative. In a study conducted to promote FV consumption among college students using class-based general nutrition course, both FV intake increased significantly to 2.9 servings over a 15-week period time [17]. The magnitude of achievement for FV was similar as demonstrated in this study. Unfortunately, no effect was seen for trend of vegetables intake through the implementation of multimodal NEI in the present study. This result was consistent with a cluster-randomized trial conducted among smokers in public housing, showing more of an increase in fruit intake than vegetable consumption [21]. It was notable that both IG and CG participants readily had achieved half of the three servings per day recommendation for vegetables intake at baseline and it was difficult to observe an increase of intake in a short duration of time as designed in this study. 

The multimodal NEI delivered in this study also found to be effective in cutting down the frequency of processed food, that is, burgers, nuggets, sausages, and French fries consumption into half relative to baseline intake pattern. These foods were seen to be substituted with healthier choice of protein from fish and egg which showed significant increase over 10-week time among IG participants. On the contrary, although baseline intakes were in acceptable range, no changes were observed in consumption of beverages loaded with sugar that is, carbonated drinks, beverages with sweetened condensed milk and beverages with added sugar. Changing processed food or fast food and energy-dense beverages intake among early adults was commonly challenging and not many have reported such findings although this is a major dietary concern among this age group [16]. Exceptionally, in a study where New York middle-school students received lessons that used science inquiry investigations to enhance motivation for action, and social cognitive and self-determination theories to increase personal agency and autonomous motivation to take action on their diet reported consumption of considerably fewer sweetened drinks and packaged snacks and smaller sizes of fast food among their participants in intervention arm [29]. The study found that it was encouraging when youth appeared to be responsive to an approach that helps them understand that they have choices, can exert control, and can make changes in their own eating as well as their personal food environments to enhance their health and to help their bodies do what they want them to do, somewhat comparable with the approach used in the current multimodal NEI. 

Intake of milk, and dairy products, one of the main sources of calcium from diet for bone health, was also increased three times higher from baseline after 10 weeks of intervention in the present study. This agrees with a class-based nutrition intervention study among college students conducted earlier which found a positive change in milk intake pattern [30]. The total milk consumption, specifically fat free milk, has increased in females and male students changed milk choice favouring skimmed milk over low fat milk in the latter study showing that there was a difference on preferences towards milk types based on gender. However, it should be noted that, even after the intervention, milk intake was still much lower than the recommended levels, two cups per day, although total milk consumption increased after the intervention in both studies [24]. This was not surprising as it was consistent with numerous researches which reported low intake of milk consumption among young adults in this region [31, 32]. One possible explanation was that young adults tend to believe they were at low risk of developing health-related problems specifically associated with nutrition and dairy intake. Conversely, a study using web-based NEI incorporating e-mail messages, posted information, and behaviour checklists with tailored feedback however only successfully increased self-regulatory strategies and self-efficacy for consuming three servings per day of dairy products, but not in outcome expectations or consumption of dairy products in five-week time [33]. Besides shorter duration of intervention, compared to other studies, the changes observed in self-efficacy may not have been large enough to produce concomitant changes in outcome expectations. Therefore, an intervention which targets both increasing self-efficacy and measureable dietary changes would be needed to improve milk and dairy products intake in meeting dietary recommendation.

These findings are important because increasing fruits, fish, egg, milk, dairy products, and less frequent consumption of processed foods would eventually increase dietary fibre, calcium, vitamin A, C, E, and D, omega-3 fatty acids and reduce trans fat and sodium intake, all which were known to be of problem in today's diet and health behaviour among 18- to 24-year-old university students [4–7, 11, 12]. Additionally, the current investigation consistent with previous research has proven that an increase in nutrition knowledge directly predicted short-term positive behavioural changes [5, 17, 27]. 

There were several reasons why a promising outcome was observed among the current study participants. The use of easy to understand key messages and achievable targets as the main content of this multimodal NEI should have contributed to the positive outcome seen in this study. Development process of the key messages was detailed out elsewhere [24, 34]. Briefly, the key messages were prepared by a group of experts in nutrition and public health who reviewed current scientific evidence-based recommendations and dietary habits of Malaysian. The draft key messages and recommendation were deliberated through several rounds of discussion with the members of panel and pretested in a sample of the community and finally all stakeholders and external reviewers were given the opportunity to comment on the final draft. Besides, the lecture which focused on how to achieve the targets for each key message was found to be interesting by the participants of this study, similar to the findings during acceptability study [25]. On top of the 1-hour lecture, take-home messages using easy to read brochures might have also helped the participants in the IG recall and refer to the NEI delivered. 

Another possible explanation for the success of the intervention might be attributed to the use of innovative technology as part of multimodal NEI to reach the study participants through text messaging. Text messaging was fully integrated into lives of university students along with other social media. Survey findings confirmed that the mainstay of the local mobile telephone subscriber base was young adults in the 20- to 24-year old age group accounting for 17.3% of the total respondents [35]. In the current study, text messages were designed to encourage and reinforce the NEI provided throughout the intervention period after the delivery of conventional lecture and brochures. The use of positive, simple, few in number, and culturally appropriate text messages delivered in this study has also shown some encouraging effect. 

This study has several inevitable limitations. Firstly, all measures were self-reported which were highly dependent on the participants' memory, honesty, and truthfulness in answering the questions. Unfortunately, nutrition education might result in “improvements” of reported intake without changes in real intake. Whether the positive effects of dietary outcomes would persist or being attenuated in the long run was beyond the scope of this study. Thus, future research should be directed toward longitudinal studies to examine long-term effect of multimodal NEI on changes in dietary intake. The sample of participants was also rather unbalanced between genders due to difficulty in recruiting males compared to females, a common trend in the universities in Malaysia and the same trend is believed to have also occurred elsewhere [36, 37]. Furthermore, the percentage of drop out was quite high among males compared to female respondents (male = 16.1%; female = 7.8%). 

However, several study strengths which included ensuring adequate randomization, a good adherence towards multimodal NEI, and generally strong program satisfaction were observed in this study with all participants in the IG, who agreed they would recommend the multimodal NEI to their friends and family members. This study was also focused on obtaining accurate dietary data using dedicated questionnaires and methods to administer them as well as the inclusion of large samples to substantiate the findings. Bias in data analysis was excluded by having only one nutritionist who was blinded to group assignments for diet history data analysis. The overall data was rechecked for consistency by another RA for quality control purposes.

## 5. Conclusion

The present study showed that multimodal NEI focusing on healthy eating promotion is an effective approach to improve dietary intakes among university students. With minimum additional manpower and financial resources in a university setting, this study also provides initial information on the use of conventional lecture, brochures, and text messaging for the delivery of effective NEI over a 10-week period which has the potential for broad reach and dissemination across university campuses. Future research is needed to determine the level of continued engagement and utilization of the multimodal platforms for NEI, as well as sustainability of dietary behaviour since even small to modest dietary level improvement disseminated on broad scale could have a positive effect on the population health.

## Figures and Tables

**Table 1:** The themes and key messages incorporated in the nutrition education module [21].

Themes	Messages
(1) Always be healthy	(i) Eat a variety of foods within your recommended intake. (ii) Maintain body weight in a healthy range. (iii) Be physically active every day.

(2) Eat moderately	(i) Eat adequate amount of rice, other cereal products (preferably whole grain) and tubers. (ii) Eat plenty of fruits and vegetables every day. (iii) Consume moderate amounts of fish, meat, poultry, egg, legumes and nuts. (iv) Consume adequate amounts of milk and milk products. (v) Drink plenty of water daily.

(3) Live the future	(i) Limit intake of foods high in fats and minimise fats and oils in food preparation. (ii) Choose and prepare foods with less salt and sauces. (iii) Consume foods and beverages low in sugar. (iv) Consume safe and clean foods and beverages. (v) Make effective use of nutrition information on food labels.

**Table 2:** Selected characteristics of study participants.

Characteristics	CG (*n* = 202)	IG (*n* = 178)
Age (year)*	19.2 ± 1.1	19.0 ± 1.2
Height (cm)*	157.8 ± 7.2	156.5 ± 7.1
Weight (kg)*	53.6 ± 12.3	51.9 ± 9.5
Waist circumference (cm)*	68.8 ± 11.2	67.6 ± 9.9
Hip circumference (cm)*	90.0 ± 10.2	89.9 ± 9.6
Gender^a^		
Male	35 (17.3)	12 (6.7)
Female	167 (82.7)	166 (93.3)
Living arrangement^a^		
Alone	2 (1.0)	9 (5.1)
With family	17 (8.4)	27 (15.2)
With friends	183 (90.6)	142 (79.8)
Academic year^a^		
First year	129 (63.9)	114 (64.0)
Second year	73 (36.1)	64 (36.0)
Funding status^a^		
Funded	149 (73.8)	130 (73.0)
Not funded	52 (25.7)	48 (27.0)
BMI classification^a^		
Underweight	46 (22.8)	48 (27.0)
Normal weight	102 (50.5)	82 (46.1)
Overweight	54 (26.7)	48 (27.0)

*Data expressed as mean ± SD

^a^Data expressed as *n* (%).

**Table 3:** Mean daily energy and macronutrient intakes in intervention (*n* = 178) and control (*n* = 202) group and ANCOVA analysis after controlling for potential confounders.

Macronutrient intakes	Mean ± SE		Adj. mean (95% CI)^a^	Adj. mean diff. (95% CI)^b^	*F*-stat (df)	*P* value^a^	Adjusted effect size (Cohen's *d*)
	Baseline	After 10-weeks					
Energy (kcal)				68 (20, 116)	7.6 (1, 376)	0.006	0.28 (S)
Intervention	1563 ± 20	1705 ± 20	1706 (1671, 1741)
Control	1564 ± 19	1638 ± 20	1638 (1605, 1671)
% Carbohydrate of energy				0.6 (−0.5, 1.8)	1.2 (1, 376)	0.27	0.11 (N)
Intervention	51.9 ± 0.4	54.6 ± 0.4	54.7 (53.9, 55.5)
Control	51.6 ± 0.4	54.0 ± 0.4	54.1 (53.3, 54.8)
% Protein of energy				−0.2 (−0.7, 0.3)	0.8 (1, 376)	0.38	0.09 (N)
Intervention	13.9 ± 0.1	13.6 ± 0.1	13.6 (13.3, 13.9)
Control	13.8 ± 0.1	13.8 ± 0.1	13.8 (13.5, 14.1)
% Fat of energy				−0.4 (−1.4, 0.6)	0.6 (1, 376)	0.42	0.08 (N)
Intervention	34.0 ± 0.3	31.7 ± 0.3	31.7 (31.0, 32.5)
Control	34.4 ± 0.3	32.1 ± 0.3	32.1 (31.4, 32.8)
Calcium				78.0 (53.5, 102.4)	39.3 (1, 376)	<0.001	0.65 (M)
Intervention	312.6 ± 7.5	376.5 ± 9.4	378.4 (360.5, 396.2)
Control	331.4 ± 7.3	300.6 ± 8.2	300.4 (283.7, 317.1)
Iron				0.2 (−1.8, 2.1)	0.0 (1, 376)	0.84	0.02 (N)
Intervention	19.4 ± 0.7	20.1 ± 0.8	20.1 (18.7, 21.5)
Control	19.6 ± 0.6	19.9 ± 0.6	19.9 (18.6, 21.2)
Vitamin C				57.6 (45.0, 70.1)	81.8 (1, 376)	<0.001	0.93 (L)
Intervention	55.9 ± 4.7	101.9 ± 5.6	100.5 (91.3, 109.6)
Control	44.7 ± 3.9	42.0 ± 3.6	42.9 (34.4, 51.5)
Thiamine				0.1 (0.0, 0.2)	4.5 (1, 376)	0.03	0.22 (S)
Intervention	1.0 ± 0.0	1.1 ± 0.0	1.1 (1.0, 1.1)
Control	1.0 ± 0.0	1.0 ± 0.0	1.0 (0.9, 1.0)
Riboflavin				0.1 (0.0, 0.2)	1.8 (1, 376)	0.18	0.14 (N)
Intervention	1.6 ± 0.0	1.6 ± 0.0	1.6 (1.5, 1.8)
Control	1.6 ± 0.0	1.6 ± 0.0	1.6 (1.5, 1.6)
Niacin				−0.3 (−1.2, 0.6)	0.5 (1, 376)	0.49	0.07 (N)
Intervention	15.2 ± 0.3	14.7 ± 0.3	14.6 (13.9, 15.3)
Control	14.6 ± 0.3	14.9 ± 0.3	14.9 (14.3, 15.5)

^a^Adjusted mean using ANCOVA after controlling for weight, waist, hip and baseline for each variable

^b^Bonferroni adjustment for 95% CI for difference; Adjusted effect size (N: Negligible, S: Small, M: Medium, L: Large); SE: Standard error of the mean.

**Table 4:** Mean daily servings of food intakes in intervention (*n* = 178) and control (*n* = 202) group and ANCOVA analysis after controlling for potential confounders.

Food (serving/day)	Mean ± SE		Adj. mean (95% CI)^a^	Adj. mean diff. (95% CI)^b^	*F*-stat (df)	*P* value^a^	Adjusted effect size (Cohen's *d*)
	Baseline	After 10-weeks					
Rice				−0.7 (−1.5, 0.1)	2.9 (1, 376)	0.09	0.18 (N)
Intervention	1.70 ± 0.04	1.70 ± 0.04	11.9 (11.3, 12.5)				
Control	1.70 ± 0.04	1.80 ± 0.04	12.6 (12.0, 13.2)				
Bread				−0.5 (−1.4, 0.4)	1.3 (1, 376)	0.25	0.11 (N)
Intervention	0.73 ± 0.05	0.76 ± 0.05	5.3 (4.7, 6.0)				
Control	0.77 ± 0.05	0.85 ± 0.05	5.9 (5.2, 6.5)				
Noodles				−0.1 (−0.7, 0.5)	0.0 (1, 376)	0.83	0.02 (N)
Intervention	0.32 ± 0.04	0.33 ± 0.04	2.2 (1.7, 2.6)				
Control	0.26 ± 0.03	0.31 ± 0.03	2.2 (1.8, 2.7)				
Biscuits				0.6 (−0.4, 1.5)	1.5 (1, 376)	0.22	0.13 (N)
Intervention	0.61 ± 0.04	0.76 ± 0.05	5.4 (4.7, 6.0)				
Control	0.65 ± 0.04	0.69 ± 0.05	4.8 (4.2, 5.4)				
Cereals				−0.1 (−0.5, 0.2)	0.5 (1, 376)	0.48	0.07 (N)
Intervention	0.07 ± 0.02	0.08 ± 0.02	0.6 (0.3, 0.8)				
Control	0.08 ± 0.02	0.10 ± 0.02	0.7 (0.5, 1.0)				
Fruit and 100% fruit juice				5.8 (4.6, 6.9)	100.2 (1, 376)	<0.001	1.03 (L)
Intervention	0.40 ± 0.05	1.16 ± 0.08	8.1 (7.2, 8.9)				
Control	0.35 ± 0.04	0.32 ± 0.04	2.3 (1.5, 3.1)				
Vegetables				0.8 (−0.2, 1.8)	2.3 (1, 376)	0.12	0.16 (N)
Intervention	1.39 ± 0.06	1.45 ± 0.06	10.1 (9.3, 10.8)				
Control	1.31 ± 0.06	1.31 ± 0.06	9.3 (8.6, 10.0)				
Meat				0.0 (−0.5, 0.5)	0.0 (1, 376)	0.95	0.01 (N)
Intervention	0.21 ± 0.03	0.16 ± 0.03	1.2 (0.8, 1.5)				
Control	0.23 ± 0.03	0.17 ± 0.03	1.2 (0.8, 1.5)				
Poultry				−0.2 (−1.2, 0.8)	0.1 (1, 376)	0.73	0.04 (N)
Intervention	1.60 ± 0.06	1.64 ± 0.06	11.4 (10.7, 12.2)				
Control	1.51 ± 0.05	1.65 ± 0.05	11.6 (10.9, 12.3)				
Fish				0.8 (0.3, 1.4)	8.3 (1, 376)	0.004	0.30 (S)
Intervention	0.24 ± 0.03	0.35 ± 0.04	2.5 (2.1, 2.9)				
Control	0.24 ± 0.02	0.24 ± 0.03	1.7 (1.3, 2.0)				
Egg				0.3 (0.0, 0.6)	4.5 (1, 376)	0.03	0.22 (S)
Intervention	0.06 ± 0.01	0.13 ± 0.02	0.9 (0.7, 1.1)				
Control	0.07 ± 0.01	0.08 ± 0.01	0.6 (0.4, 0.8)				
Nuts and legumes				0.0 (0.0, 0.1)	0.1 (1, 376)	0.70	0.04 (N)
Intervention	0.01 ± 0.01	0.01 ± 0.01	0.0 (0.0, 0.1)				
Control	0.01 ± 0.00	0.00 ± 0.00	0.0 (0.0, 0.1)				
Milk				1.2 (0.7, 1.7)	22.8 (1, 376)	<0.001	0.49 (S)
Intervention	0.08 ± 0.02	0.26 ± 0.03	1.9 (1.5, 2.2)				
Control	0.09 ± 0.02	0.09 ± 0.02	0.6 (0.3, 1.0)				
Dairy products				0.5 (0.2, 0.8)	8.2 (1, 376)	0.005	0.30 (S)
Intervention	0.11 ± 002	0.13 ± 0.02	0.9 (0.7, 1.2)				
Control	0.05 ± 0.01	0.06 ± 0.01	0.4 (0.2, 0.7)				
Carbonated drinks				−0.1 (−0.3, 0.1)	1.0 (1, 376)	0.32	0.10 (N)
Intervention	0.08 ± 0.02	0.03 ± 0.01	0.2 (0.1, 0.4)				
Control	0.04 ± 0.01	0.04 ± 0.01	0.3 (0.2, 0.5)				
Beverages with added sugar				0.4 (−0.4, 1.2)	0.9 (1, 376)	0.35	0.10 (N)
Intervention	0.37 ± 0.04	0.52 ± 0.05	3.6 (3.0, 4.2)				
Control	0.37 ± 0.04	0.46 ± 0.04	3.2 (2.7, 3.8)				
Beverages with sweetened condensed milk				−0.3 (−1.3, 0.7)	0.3 (1, 376)	0.59	0.06 (N)
Intervention	1.02 ± 0.04	0.81 ± 0.06	5.7 (4.9, 6.4)				
Control	0.94 ± 0.04	0.85 ± 0.05	5.9 (5.2, 6.6)				
Processed foods				−1.3 (−2.0, −0.7)	15.6 (1, 376)	<0.001	0.41 (S)
Intervention	0.48 ± 0.04	0.26 ± 0.03	1.8 (1.3, 2.3)				
Control	0.45 ± 0.04	0.45 ± 0.04	3.1 (2.7, 3.6)				
Deep fried snacks				−0.5 (−1.0, 0.0)	3.5 (1, 366)	0.06	0.19 (N)
Intervention	0.21 ± 0.03	0.13 ± 0.02	0.9 (0.6, 1.3)				
Control	0.28 ± 0.03	0.21 ± 0.03	1.4 (1.1, 1.8)				
Sweet dessert				0.0 (−0.6, 0.5)	0.0 (1, 376)	0.95	0.00 (N)
Intervention	0.15 ± 0.02	0.18 ± 0.03	1.3 (0.9, 1.7)				
Control	0.15 ± 0.02	0.18 ± 0.03	1.3 (0.9, 1.7)				

^a^Adjusted mean using ANCOVA after controlling for weight, waist, hip and baseline for each variable

^b^Bonferroni adjustment for 95% CI for difference

Adjusted effect size (N: Negligible, S: Small, L: Large)

SE: Standard error of the mean.
